# Comparison of the Incidence of Nd:YAG Laser Capsulotomy Based on the Type of Intraocular Lens

**DOI:** 10.3390/medicina59122173

**Published:** 2023-12-14

**Authors:** Yuri Lee, Jae Suk Kim, Bum Gi Kim, Je Hyung Hwang, Min Ji Kang, Jee Hye Lee

**Affiliations:** Department of Ophthalmology, Sanggye Paik Hospital, Inje University College of Medicine, Seoul 01757, Republic of Korea; s4651@paik.ac.kr (Y.L.); s5074@paik.ac.kr (J.S.K.); s5696@paik.ac.kr (B.G.K.); s5535@paik.ac.kr (J.H.H.); s5589@paik.ac.kr (M.J.K.)

**Keywords:** intraocular lens, Nd:YAG laser, posterior capsule opacity

## Abstract

*Background and Objectives*: Posterior capsular opacification (PCO) is the most common long-term complication of successful cataract surgery and can cause visual impairment. We aimed to investigate the effects of intraocular lens (IOL) characteristics on PCO by comparing the incidence of neodymium-doped yttrium aluminum garnet (Nd:YAG) laser capsulotomy for different types of intraocular lenses. *Materials and Methods*: A retrospective analysis was performed on 2866 eyes that underwent cataract surgery between January 2010 and December 2017, with at least 5 years of follow-up. The IOLs used for surgery were the hydrophobic lenses SN60WF (Alcon, Fort Worth, TX, USA), ZCB00 (Johnson & Johnson Vision, Santa Ana, CA, USA), and MX60 (Bausch & Lomb, Rochester, NY, USA), and the hydrophilic lens MI60 (Bausch & Lomb, Rochester, NY, USA). We analyzed the incidence of Nd:YAG laser capsulotomy according to the type of IOL used. *Results*: The incidence of Nd:YAG laser capsulotomy was significantly higher with MI60 lenses (31.70%, 175/552 eyes) compared to SN60WF (7.90%, 113/1431 eyes), ZCB00 (10.06%, 64/636 eyes), and MX60 (10.57%, 13/123 eyes; *p* < 0.001) lenses. The incidence of Nd:YAG laser capsulotomy was significantly lower with the hydrophobic IOLs (8.68%, 190/2190 eyes) than with the hydrophilic IOL (31.70%, 175/552 eyes; *p* < 0.001). Over time, the rate of increase in the cumulative number of Nd:YAG laser capsulotomy cases was the highest with MI60. The cumulative rate of Nd:YAG laser capsulotomy during the first 3 years was 4.90% with SN60WF (70/1431 eyes), 6.76% with ZCB00 (43/636 eyes), 8.94% with MX60 (11/123 eyes), and 26.10% with MI60 (144/552 eyes) lenses. *Conclusions*: The incidence of PCO is influenced by the material of the IOLs. The hydrophilic IOL was associated with a higher rate of Nd:YAG laser capsulotomy than the hydrophobic IOLs, with a shorter time to Nd:YAG laser capsulotomy.

## 1. Introduction

Age-related cataracts are the major cause of blindness, and cataract surgery is the most frequently performed ophthalmologic surgery worldwide [1]. The proportion of patients who have undergone cataract surgery is increasing as a result of a population increasing in both size and age [2]. Although cataract surgery is relatively safe and effective, it is impossible to remove all lens epithelial cells from the capsule during surgery. As a result, the remaining lens epithelial cells proliferate and migrate over time, causing posterior capsular opacification (PCO). PCO is the most common long-term complication of successful cataract surgery and can cause visual impairment. When PCO occurs in the central 3 mm zone of the posterior capsule, it affects visual acuity and reduces contrast sensitivity [3].

In recent years, the incidence of PCO has declined dramatically due to improvements in cataract surgical techniques, such as the development of phacoemulsification techniques that can be performed through small incisions. In cataract surgery, effective hydrodissection, cleaning of cortical material, and removal of equatorial epithelial lens fibers helps prevent PCO [4]. Also, sealed-capsule irrigation [5] and intraocular lens (IOL) rotation in the capsular bag [6] have also been reported to be effective in preventing PCO. Additionally, the materials, designs, and shapes of the IOLs have contributed to the reduction in PCO. Silicone and acrylic IOLs are associated with a lower incidence of PCO than polymethylmethacrylate (PMMA) or hydrogel IOLs [7]. Moreover, IOLs with square optic edges are linked to lower rates of PCO than IOLs with rounded edges, as the sharp edges impede the proliferation of lens epithelial cells [8].

The standard treatment for PCO that impairs vision is a short-pulsed (1064 nm frequency), high-power solid laser procedure known as neodymium-doped yttrium aluminum garnet (Nd:YAG) laser capsulotomy. The laser optically destroys tissues, which allows the selective resection or destruction of intraocular tissues. The indication for Nd:YAG laser capsulotomy is PCO that causes the patient discomfort affecting their visual acuity and that it interferes with the visualization of the fundus [4]. Ocular hypertension, cystoid macular edema, and retinal detachment have been reported as the most common complications after Nd:YAG laser posterior capsulotomy. Also, the physical energy of the Nd:YAG laser may cause IOL dislocation or damage to the IOL [9]. Nevertheless, this method is noninvasive, convenient, and generally considered to be safe.

This study aimed to investigate how the characteristics of IOLs affect PCO by comparing the incidence of Nd:YAG laser capsulotomy in patients implanted with hydrophilic and hydrophobic IOLs in small-incision cataract surgery.

## 2. Materials and Methods

### 2.1. Database and Study Design

The medical records for 3689 eyes that underwent cataract surgery at Inje University Sanggye Paik Hospital between January 2010 and December 2017 were analyzed. A total of 2866 eyes with a minimum of 5 years of follow-up were retrospectively studied. Patients who underwent only uneventful phacoemulsification and intraocular lens implantation were enrolled. Only patients with one-piece acrylic IOLs were included.

### 2.2. Participant Selection

Patients who underwent ciliary sulcus insertion or IOL scleral fixation and patients with capsular tension ring implantation were excluded. Patients with three-piece IOL, PMMA, or silicone-type IOL implantation were excluded. Patients with cataracts secondary to inflammation, cataracts with Down syndrome, traumatic cataract, mature cataract, and atopic cataract were excluded. Patients with zonular weakness caused by pseudoexfoliation syndrome, trauma, or connective tissue disorders were excluded. Patients who underwent pars plana vitrectomy, scleral buckling, or glaucoma surgery were excluded.

The other exclusion criteria were (1) patients with poor general condition unable to visit for surgery or postoperative follow-up; (2) any history of retinal vascular diseases, including uncontrolled diabetic retinopathy and retinal artery or vein occlusion; (3) any history of inflammatory diseases, including anterior, intermediate, or posterior uveitis, and acute retinal necrosis; (4) any history of corneal diseases, including corneal opacity, Avellino dystrophy, corneal verticillata, corneal ulcer, and other corneal diseases that could affect visual acuity; and (5) any history of macular diseases, including age-related macular degeneration, macular hole, vitreomacular traction syndrome, grade 2 epiretinal membrane, and macular telangiectasia.

### 2.3. Surgical Technique

Preoperatively, cataract grading was performed by experienced ophthalmologists according to the Lens Opacities Classification System (LOCS) III standards [10]. The grades of nuclear opalescence (NO), the cortical opacities (C), and the posterior subcapsular cataract (P) scores were recorded. Prior to the surgery, measurements were taken for axial length, corneal curvature, and other parameters using AL-Scan^®^ (Nidek, Co., Ltd., Aichi, Japan). For unavailable cases, A-scan (PAC SCAN 300A; Sonomed Escalon, New Hyde Park, NY, USA) was used for axial length measurement.

All surgeries were performed by experienced surgeons, and the procedure was as follows: After applying 0.5% proparacaine hydrochloride (Alcaine, Alcon Laboratories, Fort Worth, TX, USA) for topical anesthesia, a clear corneal incision was created on the superior or temporal limbus. The ophthalmic viscoelastic device (OVD) was injected into the anterior chamber, and continuous curvilinear capsulorhexis was performed. Hydrodissection and hydrodelineation were performed, and the nucleus was subsequently removed through phacoemulsification using ultrasound (Infinity Vision System or Centurion Vision System, Alcon, Fort Worth, TX, USA). Any remaining cortical material was thoroughly removed through irrigation and aspiration (I&A). After injecting the OVD into the anterior chamber, the IOL was inserted into the capsule, and any residual OVD was removed using the I&A device. The surgical incision was not sutured but left to self-seal by inducing swelling with a balanced salt solution. The following 1-piece IOLs were inserted in the capsule: SN60WF (Alcon, Fort Worth, TX, USA), ZCB00 (Johnson & Johnson Vision, Santa Ana, CA, USA), MX60 (Bausch & Lomb, Rochester, NY, USA), and MI60 (Bausch & Lomb, Rochester, NY, USA).

### 2.4. Postoperative Follow-Up

Following the cataract surgery, the patients were observed in an outpatient setting. The patients were followed up on postoperative days 1 and 7, and at 1, 3, and 6 months. If there were no abnormalities at the 6-month follow-up, regular follow-ups were performed every 6 months thereafter. The corrected distance visual acuity (CDVA) and intraocular pressure were obtained, and a slit lamp examination and a fundus exam were performed at every follow-up visit. The refractive error measured using the autorefractor was recorded at every follow-up visit. All patients were examined using spectral-domain optical coherence tomography (Spectralis; Heidelberg Engineering, Heidelberg, Germany) at 1 month postoperatively and were excluded from the analysis if pseudophakic cystoid macular edema was observed.

**Figure 1:**
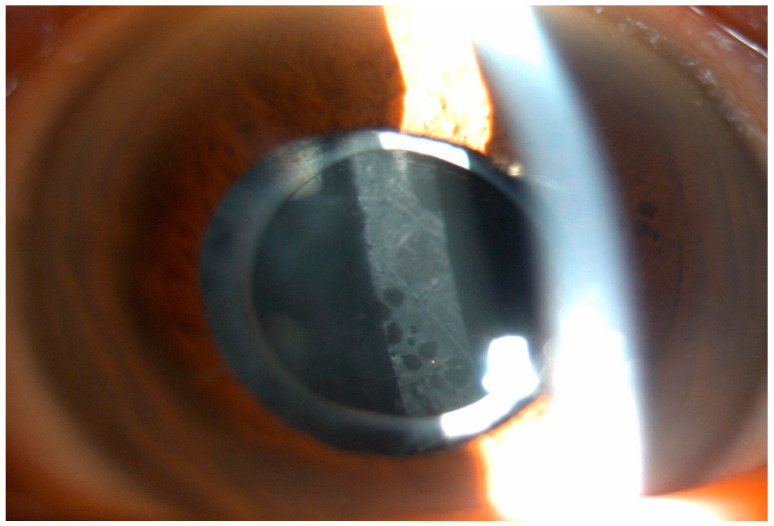
Slit-lamp photography of posterior capsular opacification.

If the patient developed discomfort due to visual impairment, experienced reduced visual acuity by at least three lines from their best-corrected vision without any other factors causing decreased visual acuity, and exhibited clear PCO on slit-lamp microscopy (Figure 1), Nd:YAG laser capsulotomy was performed. Experienced ophthalmologists performed the capsulotomy using an Ophthalmic YAG laser (YC-1800, NIDEK Co., Ltd., Aichi, Japan) after topical anesthesia. The intraocular pressure was measured before and 1 h after Nd:YAG laser capsulotomy to check the possibility of ocular hypertension.

The time point at which the Nd:YAG laser was performed was considered the time point of PCO development, and the median time for Nd:YAG laser capsulotomy was compared for each IOL group. The incidence of Nd:YAG laser capsulotomy was compared based on the type of IOL, and the correlation of Nd:YAG laser capsulotomy with sex, age at the time of surgery, and IOL diopter power was analyzed. The cumulative rate of Nd:YAG laser capsulotomy in a given time period was evaluated.

### 2.5. Statistical Analysis

The demographic parameters are presented as the mean and standard deviation. To compare the characteristics between the IOL groups, we employed one-way analysis of variance and the chi-square test. Analysis of variance was conducted to assess age, IOL diopter, and the median time for Nd:YAG laser capsulotomy in each IOL group. To compare the type of IOL between the non-capsulotomy and Nd:YAG capsulotomy groups, we used the chi-square test. Logistic regression was performed for comparative analyses of age and IOL diopter between the two groups. Statistical analyses were performed using the SPSS version 18.0 (SPSS Inc., Chicago, IL, USA). A *p*-value < 0.05 was considered statistically significant.

### 2.6. Ethics

This study was approved by the Institutional Review Board (IRB) of the Inje University Sanggye Paik Hospital (approval number: SG PAIK 2021-11-008). The requirement for written informed consent was waived due to the retrospective nature of this study. This study conformed to the Declaration of Helsinki for studies conducted in humans.

## 3. Results

Of the 2866 eyes with a minimum of 5-year follow-ups, a total of 2742 eyes were included in the final analysis after excluding cases that met the exclusion criteria: 1431 eyes (male: 483, female: 948, mean age: 68.95 ± 11.03 years, mean diopter power: 20.46 ± 3.51 D) in the SN60WF group; 636 eyes (male: 191, female: 445 female, mean age: 69.53 ± 10.15 years, mean diopter power: 20.96 ± 3.22 D) in the ZCB00 group; 123 eyes (male: 53, female: 70, mean age: 71.24 ± 9.66 years, mean diopter power: 19.05 ± 6.63 D) in the MX60 group; and 552 eyes (male: 254, female: 298, mean age: 69.20 ± 10.95 years, mean diopter power: 20.55 ± 3.19 D) in the MI60 group. There were no significant differences in age among the four groups (*p* = 0.124), but there were significant differences in sex and IOL diopter power (*p* < 0.001). The median time for Nd:YAG laser capsulotomy was 35.79 ± 18.00 months in the SN60WF group, 33.56 ± 14.57 months in the ZCB00 group, 30.46 ± 15.33 months in the MX60 group, and 31.41 ± 11.59 months in the MI60 group. No significant differences were found in the median time between each IOL group (Table 1).

Nd:YAG laser capsulotomy was performed in 129/981 male eyes (13.15%) and 236/1761 female eyes (13.40%), with no significant between-sex difference (*p* = 0.853) (Table 2). None of the patients experienced any post-Nd:YAG laser complications such as elevation of intraocular pressure, macular edema, or IOL dislocation. The mean LOCS III scores were 2.34 ± 0.75, 2.67 ± 0.87, and 1.97 ± 1.12 in the non-capsulotomy group; 2.65 ± 0.58, 2.45 ± 0.96, and 2.11 ± 1.31 in the Nd:YAG capsulotomy group for NO, C, and P, respectively. The mean age at the time of Nd:YAG laser capsulotomy was 69.73 ± 10.56 years in the non-capsulotomy group and 66.00 ± 11.50 years in the capsulotomy group. Logistic regression revealed that the odds for Nd:YAG laser capsulotomy were higher with younger age at the time of surgery (*p* < 0.001, odds = 0.970). The mean IOL diopter power was 20.57 ± 3.54 in the non-capsulotomy group and 20.25 ± 3.99 in the capsulotomy group. The IOL diopter power did not significantly affect the odds for Nd:YAG laser capsulotomy (*p* = 0.110) (Table 2).

Regarding the type of IOL, Nd:YAG laser capsulotomy was performed in 113/1431 eyes with the SN60WF implant (7.90%), 64/636 eyes with ZCB00 implant (10.06%), 13/123 eyes with the MX60 implant (10.57%), and 175/552 eyes with the MI60 implant (31.70%). The rate of Nd:YAG laser capsulotomy was highest in the MI60 group, and the chi-square test confirmed a significant difference in the rate of Nd:YAG laser capsulotomy according to the type and material of IOL used (*p* < 0.001) (Table 2).

The incidence of Nd:YAG laser capsulotomy was compared for each type of IOL using pairwise comparison as well, and the rate of Nd:YAG laser capsulotomy was significantly different in the MI60 group compared to those in the other IOL groups (*p* < 0.001, chi-square test). There were no significant differences in the rate of Nd:YAG laser capsulotomy among the remaining IOL groups (*p* = 0.104 in SN60WF and ZCB00 comparison; *p* = 0.297 in SN60WF and MX60 comparison; *p* = 0.865 in ZCB00 and MX60 comparison) (Table 3).

In terms of hydrophobic and hydrophilic IOLs, the rate of Nd:YAG laser capsulotomy was significantly lower with hydrophobic IOLs (SN60WF, ZCB00, MX60; 190/2190 eyes, 8.68%) than with hydrophilic IOL (MI60; 175/552 eyes, 31.70%; *p* < 0.001, chi-square test) (Table 2).

**Figure 2:**
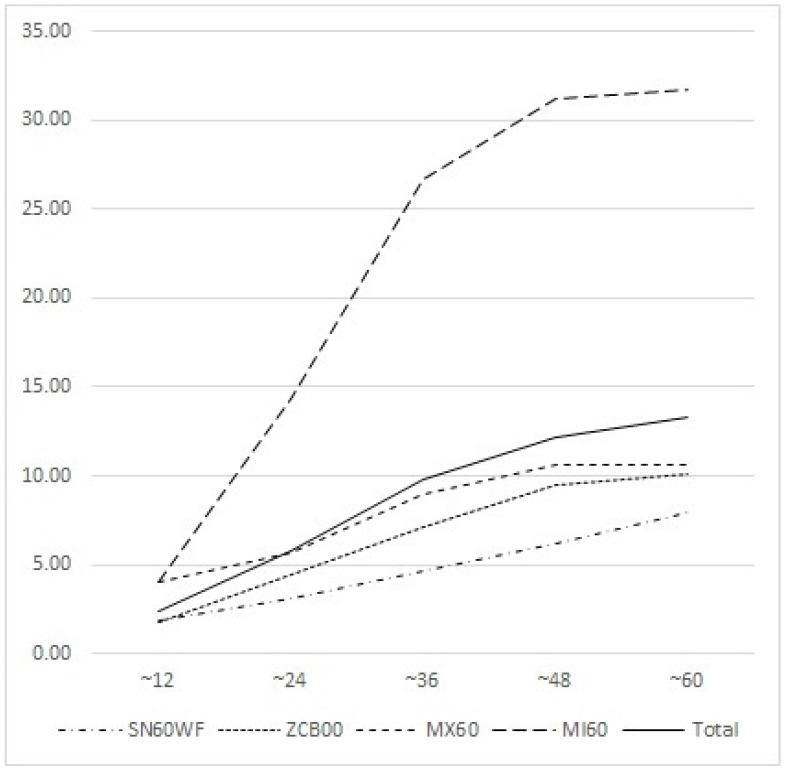
Cumulative Nd:YAG laser posterior capsulotomy rates over time. The rate of increase was steepest in MI60 compared to those of the other IOLs.

In terms of the cumulative rate of Nd:YAG laser capsulotomy during a given period, the rate of increase in the number of Nd:YAG laser capsulotomy cases was higher in the MI60 group than in the other IOL groups. The cumulative rate of Nd:YAG laser capsulotomy at 1 year was 2.02% with the SN60WF (29/1431 eyes), 1.73% with the ZCB00 (11/636 eyes), 4.07% with the MX60 (5/123 eyes), and 4.34% with the MI60 (24/552 eyes) lenses. There were no significant differences in the rates of Nd:YAG laser capsulotomy at 1 year. At 2 years, the gap between the capsulotomy rates of the MI60 and other IOLs began to widen. A dramatic increase in the number of capsulotomy cases occurred in the first 3 years after surgery. The cumulative rate of Nd:YAG laser capsulotomy at 3 years was 4.90% with the SN60WF lenses (70/1431 eyes), 6.76% with the ZCB00 lenses (43/636 eyes), 8.94% with the MX60 lenses (11/123 eyes), and 26.10% with the MI60 lenses (144/552 eyes) (Figure 2).

## 4. Discussion

PCO is the most common complication of cataract surgery, and various factors, such as surgical technique, shape, and material of the IOL, and the patient’s general state, are believed to affect its onset.

The SN60WF, ZCB00, MX60, and MI60 implants used in this study are acrylic one-piece spherical monofocal square-edged IOLs. One key difference is that SN60WF, ZCB00, and MX60 are hydrophobic, whereas MI60 is hydrophilic. Hydrophilic IOLs possess a low refractive index due to their high water content. They are flexible and can be inserted through a small incision of 1.8 mm, which makes them useful for micro-incision cataract surgery [11]. Moreover, they possess low aqueous flare with a relatively low accumulation rate of inflammatory cells on the IOL surface [12]. On the other hand, hydrophobic IOLs possess good uveal biocompatibility and are associated with a relatively low incidence of PCO.

In this study, hydrophobic IOLs were associated with a significantly lower rate of Nd:YAG laser capsulotomy than hydrophilic IOLs. The results of this study are consistent with those of previous studies evaluating the effects of hydrophilic and hydrophobic IOLs on PCO. Li et al. [13] compared the incidence of Nd:YAG laser capsulotomy after 2 years of hydrophilic or hydrophobic IOL implantation in 546 eyes and reported a lower incidence with hydrophilic IOLs (risk ratio 6.96, 95% CI, *p* < 0.00001). In a 3-year follow-up of 8293 eyes in Spain, a significant difference was observed in the rate of Nd:YAG laser capsulotomy, with 5% for hydrophobic IOLs and 21.2–31.1% for hydrophilic IOLs [14]. Zhao et al. [15] analyzed a total of 889 eyes in 11 studies through meta-analysis. The analysis revealed that hydrophobic IOLs were associated with lower Nd:YAG laser capsulotomy rates than hydrophilic lenses (odds ratio 0.38, 95% CI, *p* = 0.029). Other, previous studies from various countries have also reported consistent results [16,17,18].

The molecular and cellular reasons for the differences between hydrophobic and hydrophilic IOLs are not well studied. Hydrophobic IOLs can adhere to collagen membranes, which enables firm adherence of the optics to the posterior capsule, and fibronectin enhances the adhesion [19]. These molecular interactions may reduce the space between the IOLs and the posterior capsule and thus prevent movement of the lens epithelial cells, which may lower the risk of PCO [13]. On the other hand, hydrophilic IOLs tend to have more proliferative lens epithelial cells due to their inherent characteristics. Any lens epithelial cells remaining after surgery proliferate in the area between the posterior capsule and the IOL, thereby impeding adhesion. The proliferation of lens epithelial cells occurs more extensively in the posterior chamber of the IOL and contributes to the development of PCO. In addition, hydrophilic surface properties have been shown to promote the migration of lens epithelial cells from the equatorial region to the visual region, increasing the impact of PCO [20].

In this study, we did not analyze the design of the IOL. However, we found that not only the material but also the design of IOL had an influence on the formation of PCO. Some studies have compared hydrophilic and hydrophobic IOLs with sharp optical edges using electron microscopy and revealed that hydrophobic IOLs possess sharper edges [21]. The processing of the IOL influences the shape of the edge. Hydrophilic acrylic IOLs are processed in a dehydrated state and are subsequently rehydrated, which may blunt the edges to some degree [22]. Additionally, a study comparing PCO and Nd:YAG laser capsulotomy rates in square-edged and non-squared-edged IOLs reported that an IOL with a 360° square edge may help prevent PCO by inhibiting the migration of lens epithelial cells behind the IOL [3]. The tight wrapping of the posterior capsule around the sharp square edge creates a discontinuous sharp bend with little space between the IOL optic and the lens capsule. This prevents lens endothelial cell migration in the postoptic space and hence reduces PCO [23,24]. Following the same principle, plate-haptic-designed IOLs are associated with more PCO formation than open-loop haptic-designed IOLs [23,25,26]. However, there was no difference in the occurrence of PCO between one-piece IOLs and three-piece IOLs, although it varied depending on the IOL material [27].

The surgical technique is also associated with the risk of developing PCO. The size of the continuous curvilinear capsulorhexis (CCC) and the overlap of the anterior capsule between the CCC and the lens are associated with PCO. A large CCC causes the posterior capsule to wrinkle, promoting the migration of lens epithelial cells and causing PCO. A small CCC allows the IOL to adhere well to the capsular bag and reduces the movement of lens epithelial cells, thereby reducing the occurrence of PCO [28]. Among the studies that analyzed the occurrence of PCO according to the size of the incision, one study found that small-incision cataract surgery resulted in a lower incidence of PCO than micro-incision cataract surgery [29], but another study found that there was no difference in the occurrence of PCO [30].

Regarding the cumulative number of Nd:YAG laser capsulotomy cases by the type of IOL, the incidence of Nd:YAG laser capsulotomy was higher with MI60, a hydrophilic IOL, at all time periods, and the rate of increase was also the highest. This indicates that the time from surgery to Nd:YAG laser capsulotomy is relatively shorter with the use of a hydrophilic IOL compared to a hydrophobic IOL. Particularly, the number of Nd:YAG laser capsulotomy cases increased dramatically during the first 3 years after surgery, highlighting the importance of closely monitoring patients for the first 3 years after hydrophilic IOL implantation. Nakazawa et al. [31] reported that the incidence of PCO was 20.7% at 2 years and 28.5% at 5 years after cataract surgery. Additionally, previous studies have indicated that approximately 5–20% of patients require laser capsulotomy 3–5 years after cataract surgery [32,33].

To identify other potential factors that may influence the onset of PCO, we examined the sex, age, and diopter power of the IOL. Age was associated with Nd:YAG laser capsulotomy, whereas sex and IOL diopter power were not. The incidence of Nd:YAG laser capsulotomy increased with younger age at the time of surgery. This may be attributable to the fact that younger patients exhibit more rapid proliferation, regeneration, and migration of lens epithelial cells and are at higher risk for PCO. Previous studies also reported that younger patients had a higher incidence of PCO [26,34].

Wu et al. [35] reported that hydrophobic acrylic IOLs were associated with lower PCO scores and a lower rate of Nd:YAG capsulotomy when compared with hydrophilic IOLs, especially for Western populations, in their meta-analysis. This is significant as it represents the largest domestic study comparing the incidence of Nd:YAG laser capsulotomy based on the type of IOL used. Moreover, we investigated other potential risk factors for PCO, such as sex, age, and IOL diopter power.

One limitation of this study is that the onset of PCO was indirectly estimated based on the rate of Nd:YAG laser capsulotomy, so it is possible that not all cases of PCO were included in this study. In addition, the shapes of the four different acrylic IOLs were not identical, and this difference could have contributed to the onset of PCO. While there were three types of hydrophobic IOLs (2190 eyes) analyzed, only one type of hydrophilic IOL (552 eyes) was included in the analysis, and the substantial difference in the sample size between the two groups constitutes another limitation of this study.

Although Nd:YAG laser capsulotomy is an easy and safe technique, it may lead to complications such as macular edema, retinal detachment, damage to the IOL, dislocation of the IOL, and increased intraocular pressure. Therefore, it is crucial to reduce the number of cases of Nd:YAG laser capsulotomy by minimizing the risk for PCO.

## 5. Conclusions

The incidence of Nd:YAG laser capsulotomy is affected by the material of the IOL used, and hydrophilic IOLs are associated with a higher rate of Nd:YAG laser capsulotomy compared to hydrophobic IOLs, with a shorter time from surgery to Nd:YAG laser capsulotomy. The incidence of Nd:YAG laser capsulotomy increased with younger age, which is a risk factor for PCO. These results may provide guidance when selecting the type of IOL for cataract surgery in clinical practice.

## Figures and Tables

**Table 1:** Patient demographics.

	SN60WF	ZCB00	MX60	MI60	Total	*p*-Value
Age (years)	68.95 ± 11.03	69.53 ± 10.15	71.24 ± 9.66	69.20 ± 10.95	69.24 ± 10.76	0.124 *
Sex (M/F)	483/948	191/445	53/70	254/298	981/1761	<0.001 †
IOL diopter (D)	20.46 ± 3.51	20.96 ± 3.22	19.05 ± 6.63	20.55 ± 3.19	20.53 ± 3.60	<0.001 *
Median time for Nd:YAG laser (months)	35.79 ± 18.00	33.56 ± 14.57	30.46 ± 15.33	31.41 ± 11.59	33.10 ± 14.63	0.084 *

Values are presented as mean ± SD. IOL = intraocular lens. * Analysis of variance; † chi-square test.

**Table 2:** Rates of non-capsulotomy and Nd:YAG laser posterior capsulotomy.

	Total	Non-Capsulotomy	Nd:YAG Capsulotomy	*p*-Value
Total number	2742	2377	365	
Sex				
Male	981 (35.78)	852 (86.85)	129 (13.15)	0.853 *
Female	1761 (64.22)	1525 (86.60)	236 (13.40)	
Age (years)	69.24 ± 10.76	69.73 ± 10.56	66.00 ± 11.50	<0.001 †
IOL				
SN60WF	1431 (52.19)	1318 (92.10)	113 (7.90)	<0.001 *
ZCB00	636 (23.19)	572 (89.94)	64 (10.06)	
MX60	123 (4.49)	110 (89.43)	13 (10.57)	
MI60	552 (20.13)	377 (68.30)	175 (31.70)	
Hydrophobic	2190 (79.87)	2000 (91.32)	190 (8.68)	<0.001 *
Hydrophilic	552 (20.13)	377 (68.30)	175 (31.70)	
IOL diopter (D)	20.53 ± 3.60	20.57 ± 3.54	20.25 ± 3.99	0.110 †

Values are presented as n (%) unless otherwise indicated. Values for age and IOP diopter are presented as mean ± SD. Nd:YAG = neodymium-doped yttrium aluminum garnet; IOL = intraocular lens. * Chi-square test; † logistic regression.

**Table 3:** *p*-values of the pairwise comparisons of the rates of Nd:YAG laser posterior capsulotomy of IOLs.

	SN60WF	ZCB00	MX60	MI60
SN60WF		0.104	0.297	<0.001
ZCB00	0.104		0.865	<0.001
MX60	0.297	0.865		<0.001
MI60	<0.001	<0.001	<0.001	

Chi-square test.

## Data Availability

All data generated or analyzed during this study are included in this article. Further inquiries can be directed to the corresponding author.
